# Coupling Peptide-Based Encapsulation of Enzymes with Bacteria for Paraoxon Bioremediation

**DOI:** 10.1021/acsami.4c06501

**Published:** 2024-06-26

**Authors:** Yoav Dan, David Gurevich, Ofir Gershoni, Francesca Netti, Lihi Adler-Abramovich, Livnat Afriat-Jurnou

**Affiliations:** † Department of Oral Biology, The Goldschleger School of Dental Medicine, Faculty of Medical and Health Sciences, Tel Aviv University, Tel Aviv 6997801, Israel; ‡ The Center for Nanoscience and Nanotechnology, Tel Aviv University, Tel Aviv 6997801, Israel; § The Center for the Physics and Chemistry of Living Systems, Tel Aviv University, Tel Aviv 6997801, Israel; ∥ Migal-Galilee Research Institute, Kiryat Shmona 11016, Israel; ⊥ The Faculty of Sciences and Technology, Tel-Hai College, Upper Galilee 1220800, Israel

**Keywords:** organophosphates, phosphotriesterase, bioremediation, enzyme encapsulation, nanostructures, one-pot system

## Abstract

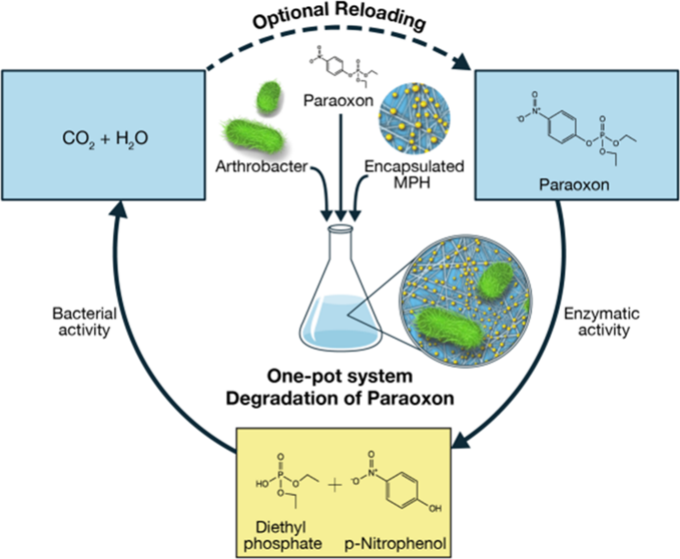

The catalytic efficiency of enzymes can be harnessed as an environmentally friendly solution for decontaminating various xenobiotics and toxins. However, for some xenobiotics, several enzymatic steps are needed to obtain nontoxic products. Another challenge is the low durability and stability of many native enzymes in their purified form. Herein, we coupled peptide-based encapsulation of bacterial phosphotriesterase with soil-originated bacteria, *Arthrobacter* sp. 4Hβ as an efficient system capable of biodegradation of paraoxon, a neurotoxin pesticide. Specifically, recombinantly expressed and purified methyl parathion hydrolase (MPH), with high hydrolytic activity toward paraoxon, was encapsulated within peptide nanofibrils, resulting in increased shelf life and retaining ∼50% activity after 132 days since purification. Next, the addition of *Arthrobacter* sp. 4Hβ, capable of degrading para-nitrophenol (PNP), the hydrolysis product of paraoxon, which is still toxic, resulted in nondetectable levels of PNP. These results present an efficient one-pot system that can be further developed as an environmentally friendly solution, coupling purified enzymes and native bacteria, for pesticide bioremediation. We further suggest that this system can be tailored for different xenobiotics by encapsulating the rate-limiting key enzymes followed by their combination with environmental bacteria that can use the enzymatic step products for full degradation without the need to engineer synthetic bacteria.

## Introduction

Xenobiotic compounds that severely affect the environment, are usually highly toxic and persistent and have limited biodegradability. Such is the case of organophosphate (OP)-based pesticides commonly used for the treatment of crops against pests.^1^ OP-based pesticides are still used in our daily environment as well as in the agriculture and food industries.^2^ However, OPs are known for their high toxicity as they irreversibly inhibit acetylcholinesterase (AChE), the enzyme responsible for the regulation of the neurotransmitter acetylcholine through its hydrolysis in the neuronal synapse.^3^ OPs can accumulate in agriculture soils and the surrounding aquatic environments, bearing various ecological as well as health impacts.^4−6^ The conventional approaches for OP degradation include chemical decontamination, such as hydrolysis, oxidation, or reduction.^7^ However, most current methods are often aggressive and not entirely safe, in terms of chemical byproducts. The degradation of both OPs and their toxic byproducts is required to avoid any possible pollution and environmental health hazards.^8,9^ Therefore, different on-site, cost-effective, eco-friendly technologies for degrading OPs into harmless compounds are being developed, including those involving microbial populations that can utilize these xenobiotics as a source of nutrients, known as bioremediation.^8,9^ This need has also motivated studies aimed at developing technologies based on recombinantly expressed and purified OP-degrading enzymes such as phosphotriesterase and organophosphorus hydrolase (OPH). Naturally or artificially designed phosphotriesterases were previously explored mostly for the decontamination and detoxification of OPs, showing encouraging results.^10−12^ The biodegradation of OP-based pesticides using enzymes is a potential strategy for the treatment of soil and aquatic contaminants as well.^2,13,14^ One such example of an OP-degrading enzyme is methyl parathion hydrolase (MPH, E.C.3.1.8.1), which belongs to the metallo-β-lactamase superfamily. MPH was shown to be a dimer with a mixed hybrid binuclear zinc center coordinated by residues His147, His149, Asp151, His152, His234, and His302.^15^ It was first identified in soil bacteria *Pseudomonas sp*. WBC-3 and isolated from soil contaminated with methyl parathion. Using 300 mg/L OP methyl parathion as its sole carbon and nitrogen source, *Pseudomonas sp*. WBC-3, is capable of degrading 15 mg/L of parathion per hour.^16^ Recombinant expression and purification of this phosphotriesterase and others have provided a promising alternative for OP decontamination. However, the application of enzymes is hampered by two main shortcomings. The first is the fact that the use of phosphotriesterases for the hydrolysis of OPs, such as paraoxon and parathion, results in the production of para-nitrophenol (PNP), which is considered a refractory pollutant capable of causing various health issues.^17,18^ Moreover, not many microorganisms in the environment can utilize OPs as a nutrients source since multiple, specialized enzymes are required for the task.^19^ Previously, it was shown that by using systems composed of OP-degrading enzymes and metal nanoparticles, chemoenzymatic systems could be obtained to further reduce the PNP into the less toxic 4-aminophenol.^20,21^ In addition, bacterial engineering (using plasmids or genome engineering) of single species was also exploited as a potential paraoxon bioremediation method.^19,22,23^ Another option that was explored is the use of a combination of two engineered bacteria, *Escherichia coli* SD2 containing a gene encoding for parathion hydrolase and *Pseudomonas putida* KT2440 pSB337 carrying the genes encoding for PNP mineralization.^24^ In both cases of bacterial species or consortium construction, using different synthetic biology approaches requires the engineering of multiple enzymatic steps and finding the optimal conditions to stably maintain functionality in a fluctuating environment. On the other hand, application of enzymes for decontamination requires adjustment of their biochemical properties, particularly their stability and durability.^25^ Generally, the widespread industrial and biological applications of most native enzymes are often obstructed by their long-term operational instability, short shelf life, and challenging recovery and reusability. Protein design and enzyme evolution,^26−29^ as well as enzyme immobilization and encapsulation, are common strategies to overcome these obstacles.^30,31^ Previously, it has been shown that immobilized or encapsulated enzymes have longer operational stability and shelf life under various conditions and are more resistant to denaturation compared to their corresponding soluble form.^31,32^ In addition, immobilized enzymes may be recycled by utilizing the physical or chemical properties of the Supporting material. For example, SsoPox, an enzyme with phosphotriesterase activity, was covalently immobilized on hydrophilic membranes and exhibited high paraoxon degradation (∼90%) and long-term stability (1 year).^33^ Enzyme encapsulation methods within a carrier provide a promising route to their stabilization. These methods typically consist of the carrier assembled in the presence of the soluble enzyme and therefore avoid the requirement of a prefabricated support material.^34^ Furthermore, the encapsulation process mostly does not affect the enzyme fold, and therefore, enzymatic activity is mainly dependent on substrate penetration.^35^ Previously, enzymes have been entrapped within different carriers including polymers, proteins, DNA, organic molecules, silica, metal–organic frameworks, and more.^36−40^ Among the various optional materials for enzyme stabilization, the use of low-molecular-weight peptides is of particular interest for industrial applications. Low-molecular-weight peptide nanostructures have unique properties, including biocompatibility, structural and chemical diversity, robustness, and large-scale synthesis, making them ideal candidate materials for engineering, medicine, and biology.^41−43^ Several peptides, such as diphenylalanine (FF) and its derivatives, have been shown to self-assemble into supramolecular structures while forming various morphologies including tubes, spheres, toroids, plates, quantum dots, and gels.^44,45^ Previously, various peptide-based nanostructures were shown to form thermally stable nanoscale-ordered structures and have retained their ultrastructure in a range of pH values and in various organic solvents, showcasing their high stability.^46^ We have recently shown that the encapsulation of a lactonase in nanospherical capsules composed of the N-tert-butoxycarbonyl-phenylalanyl-phenylalanine (BocFF) peptide resulted in the extension of the enzyme shelf life for more than 5 weeks.^32^ An additional derivative of FF, namely N-(fluorenylmethoxycarbonyl)-phenylalanyl-phenylalanine (FmocFF), has also been used to encapsulate and protect enzymes from oxygen damage within hydrogels.^47^

**Scheme 1:**
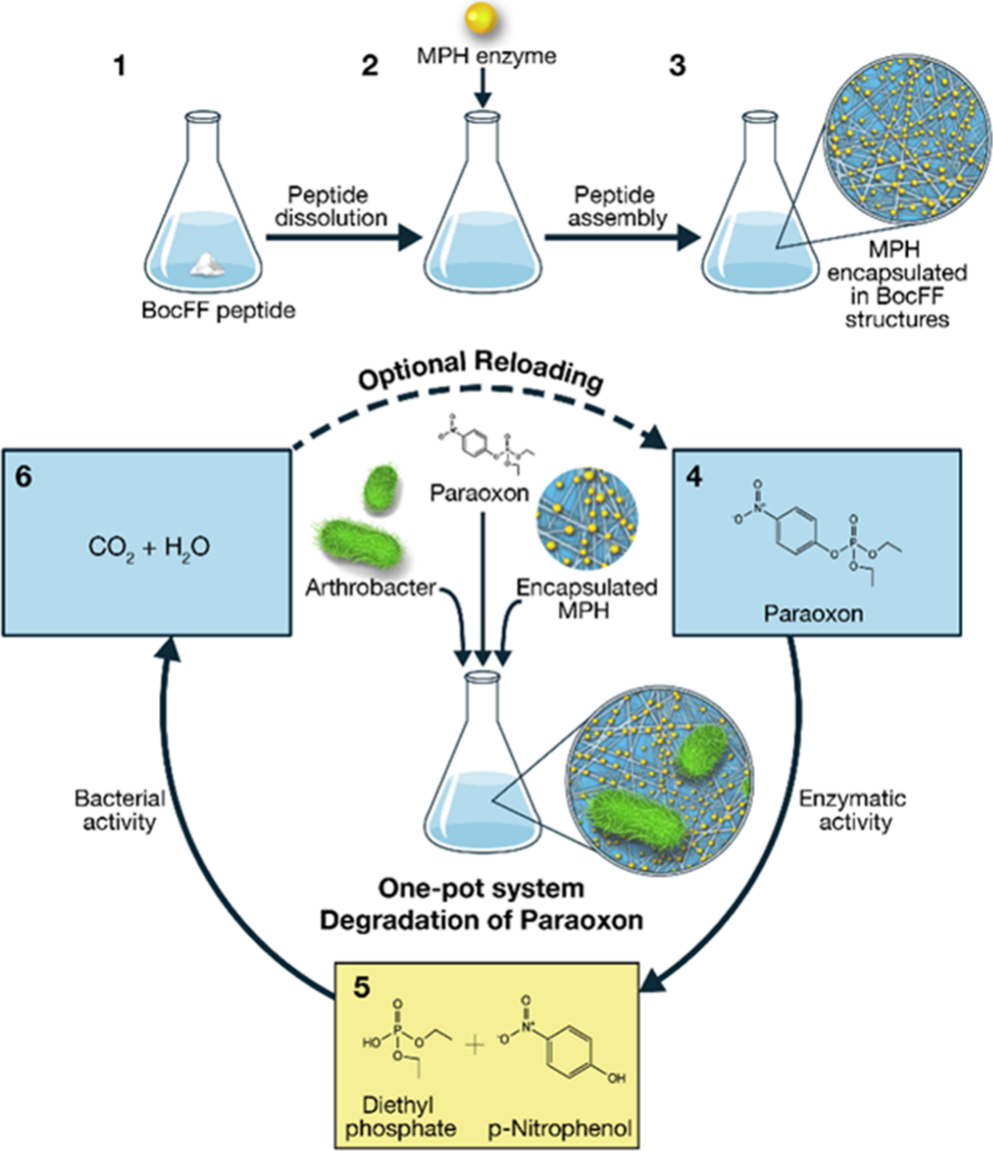
Schematic Illustration of the Reaction Cycle Mediated by the One-Pot System Assembly of BocFF-MPH Enzyme-Encapsulated Particles and PNP-Degrading Bacteria

As the use of genetically modified bacteria for environmental bioremediation is not widely accepted, here, we conjugated peptide-encapsulated MPH and naturally existing bacteria that degrade PNP. We attempted to obtain a complete degradation of paraoxon and its byproducts in a one-pot system without the need to use genetically modified organisms. Here, we applied purified MPH encapsulated within BocFF peptide nanostructures, which increased the enzyme durability, with *Arthrobacter* sp. 4Hβ, a PNP-degrading bacterial culture, to obtain an environmentally friendly one-pot system capable of detoxifying paraoxon and its byproduct (Scheme 1).

## Methods and Materials

### Cloning, Expression, and Purification of MPH

The codon-optimized synthetic gene encoding the wild-type methyl parathion hydrolase (MPH, E.C.3.1.8.1) from *Pseudomonas* sp. WBC-3 was synthesized by GenScript. MPH, GenBank accession number: AY251554, was previously shown to hydrolyze a wide range of organophosphates, with a catalytic proficiency as high as *k*_cat_/*K*_M_ of 10^6^ M^–1^ s^–1^, and its three-dimensional structure was solved.^15,48−50^ The gene was then cloned into the pET28a (+) vector at the *Nco*I and *Not*I sites to include the 6xHis-tag at the C-terminus of the protein. The resulting pET28a-MPH plasmid was used for expression and purification. Hereinafter, MPH-6xHis is referred to as MPH.

### Small-Scale Expression of MPH with Different Metals

For small-scale protein expression, 3 mL of Luria–Bertani (LB) medium containing 100 μg/mL kanamycin and 0.1 mM of either CaCl_2,_ CoCl_2,_ MnCl_2_, ZnCl_2_, or a negative control without any added metal was inoculated with a single colony of *E. coli*-BL21 (DE3) cells, freshly transformed with pET28-MPH. Cells were grown overnight at 37 °C. On the following day, the cultures were diluted 1:100 in 3 mL of the same medium and grown at 30 °C with shaking for ∼5 h. When OD_600_ reached values of 0.6–0.8, 0.4 mM isopropyl β-D-1-thiogalactopyranoside (IPTG) was added to induce expression. Following overnight incubation at 30 °C, cells were harvested by centrifugation and resuspended in 250 μL of BugBuster Protein Extraction Reagent (Merck) diluted 10-fold in activity buffer (100 mM Tris pH 8.0, 100 mM NaCl, and 0.1 mM of either CaCl_2,_ CoCl_2,_ MnCl_2,_ ZnCl_2_, or no metal as control). Lysis was then performed by incubation at 25 °C with shaking at 950 rpm for 1 h using a Thermal block shaker. After centrifugation, the supernatant was used for activity measurements. Pellets were resuspended in 250 μL of activity buffer. Sodium dodecyl sulfate–polyacrylamide gel electrophoresis (SDS–PAGE, 15%) analysis of the supernatant and pellet samples was performed. MPH activity was analyzed by monitoring the absorbance changes in 200 μL reaction volumes using 96-well plates and a microtiter plate reader (BioTeK, optical length of ∼0.5 cm) at 25 °C. Absorbance was monitored by monitoring the appearance of PNP as a result of paraoxon hydrolysis at 405 nm for 10 min with 15 s intervals between readings. The average slope (mOD/min) of the initial reaction velocity was determined using 1 μL lysate with 0.05 mM paraoxon. Reactions were performed in triplicates.

### Large-Scale Expression and Purification of MPH

Large-scale expression was performed as previously described and as outlined above.^50^ LB medium (10 mL) containing 100 μg/mL kanamycin and 0.1 mM MnCl_2_ was inoculated with a single colony of *E. coli*-BL21 (DE3) cells, freshly transformed with pET28-MPH, and grown overnight at 37 °C. The resulting culture was added to 1 L of the same medium and grown at 30 °C with shaking for ∼5 h. When OD_600_ reached values of 0.6–0.8, 0.4 mM IPTG was added to induce expression. Following overnight incubation at 30 °C, cells were harvested by centrifugation and resuspended in 25 mL of lysis buffer (100 mM Tris pH 8, 100 mM NaCl, 0.1 mM MnCl_2_, 10 mM NaHCO_3_, 0.1 mM DTT, 2 μL of Benzonaze and 1 Pierce Protease Inhibitor Mini Tablet (ThermoFisher)). The lysate was then sonicated for 3 min at an amplitude of 45 with 30 s intervals (Q700 sonicator, Qsonica, 12.7 mm tip), followed by centrifugation at 10,000 rpm for 30 min at 4 °C. After centrifugation, the supernatants were passed through Whatman filtration paper (pore size 11 μM, GE Healthcare), followed by loading on a Nickel His Trap column (GE Healthcare, 5 mL) adapted for the AKTA fast protein liquid chromatography (FPLC) system (AKTAPurifier 100, GE Healthcare). MPH was eluted with column buffer (100 mM Tris pH 8, 100 mM NaCl, 0.1 mM MnCl_2_) supplemented with 300 mM imidazole. Protein purity was estimated by 15% SDS–PAGE. Fractions containing the protein at more than 90% purity were dialyzed (Cellu Sep, Nominal MWCO:3,500) overnight in 2 L of dialysis buffer (100 mM Tris pH 8, 100 mM NaCl, 0.1 mM MnCl_2_) at 4 °C. The following day, protein concentration was determined using NanoDrop, and samples were stored at 4 °C.

### Analysis of MPH Activity toward Paraoxon

To determine the enzyme kinetic parameters, MPH activity was analyzed by monitoring absorbance changes in 200 μL reaction volumes using 96-well plates and a microtiter plate reader (BioTeK, optical length of ∼0.5 cm) at 25 °C. The hydrolysis of paraoxon was monitored by monitoring the appearance of PNP (ε = 9200 OD/M) at 405 nm. The reaction mixtures contained 0.1 mM paraoxon dissolved in DMSO (comprising less than 2% of the final mixture) and 5 nM enzyme in activity buffer (100 mM Tris pH 8, 100 mM NaCl, 0.1 mM MnCl_2_). Error ranges show the standard deviation of the data obtained from 3 independent measurements.

### Enzyme Optimum Temperature and Thermal Stability Analysis

The optimal temperature for enzymatic activity was determined by mixing purified MPH (5 nM) with paraoxon (0.1 mM) in activity buffer (100 mM Tris-HCl pH 8, 100 mM NaCl, 0.1 mM MnCl_2_), and the measurement was performed at various temperatures (0–80 °C). The product absorbance at 405 nm was measured from time point 0 until 30 min and the value at time point 0 was used as baseline and subtracted from each of the readings. The control sample was prepared under the same conditions but without the enzyme, and the values were subtracted from those of each corresponding test sample containing the enzyme. The highest reaction rate was defined as 100% activity. Each treatment was replicated three times. For thermal stability, 5 nM MPH was incubated at various temperatures (0–80 °C) for 30 min. Following incubation, the enzymatic activity with paraoxon (0.1 mM) was measured at 25 °C for 10 min in activity buffer. Spontaneous hydrolysis in samples without the enzyme was used as a control. The activity of MPH at different temperatures is shown as a percentage of the highest activity measured from an average of 3 repeats.

### Encapsulation of MPH in BocFF

Initially, BocFF (CAS 13122-90-2, purity >99%, Bachem, Switzerland) stock solution was prepared by diluting the lyophilized BocFF peptide powder in the enzyme activity buffer (100 mM Tris pH 8, 100 mM NaCl, 0.1 mM MnCl_2_) and heating the solution to 100 °C, while stirring until complete dissolution of the peptide was achieved. The solution was then aliquoted and left to slowly cool in order to allow the self-assembly of the peptide nanostructures. Once a temperature of 25 °C was reached, the enzyme stock solution was introduced. The purified enzyme was introduced only after a decrease in the solution temperature to avoid denaturation of the enzyme. The final concentrations in the samples were 4 mg/mL peptide and 1 μM enzyme.

### Powder X-ray Diffraction (PXRD)

BocFF and BocFF-MPH samples were prepared as outlined above, left to assemble for ∼72 h, then frozen at −80 °C, and dried using a lyophilizer. The sample powder data was collected with a Bruker D8-Discover diffractometer equipped with a Linxeye-XE linear detector using Bragg–Brentano geometry. Data was collected at room temperature using a sealed X-ray tube with the radiation Cu Ka1 (l = 1.54056 Å). Control samples consisted of a dried enzyme buffer solution.

### Gold Nanoparticles Labeling and Transmission Electron Microscopy (TEM)

MPH was conjugated to gold nanoparticles (NPs) using BioReady 40 nm NHS Gold kit (Merck) according to the manufacturer’s instructions. In short, before the reaction, the MPH enzyme stock solution was washed with phosphate buffer (NaHPO_4,_ 10 mM, pH 7.4) using a Millipore Amicon Ultra 0.5 mL centrifugal filter (10 kDa cutoff). Conjugation was then performed by adding 5 μg of MPH to 1 mL of gold NPs suspended in reaction buffer (NaHPO_4,_ 5 mM, pH 7.4, 0.5% PEG 20 kDa). The conjugation process was performed for 1 h at room temperature. Upon completion, 5 μL of quencher (50% w/v hydroxylamine) was added, and the sample was incubated for an additional 10 min. The AU_NPs_ -MPH was then purified and washed with reaction buffer using a Millipore Amicon Ultra 0.5 mL centrifugal filter and resuspended to 10 OD using diluent solution (0.5X PBS pH 8, 0.5% BSA, 0.5% Casein, 1% Tween 20, 0.05% Sodium Azide). The conjugation process was validated by absorbance spectrum measurements using a plate reader (TECAN Infinite M200PRO). Encapsulation process was then performed as outlined above to obtain samples with 0.1 OD of AU_NPs_-MPH. TEM samples were subsequently prepared by drop-casting 7.5 μL of sample solutions onto 400 mesh copper grids and were allowed to absorb. After 1 min, excess liquids were removed using a blotting paper, and negative staining was performed by applying 7.5 μL of UranylLess staining solution (Bar Naor LTD) for 1 min, followed by blotting of excess liquid. Samples were imaged using a JEM 1400plus electron microscope operating at 80 kV. ImageJ software was used to analyze the images and calculate the fiber diameter.

### MPH Fluorescent Labeling and Confocal Microscopy Analysis

MPH stock solution was centrifuged against phosphate buffer (NaHPO_4,_ 100 mM, pH 8) by using an Amicon Ultra 15 mL centrifugal filter (Merck; 10 kDa cutoff). MPH was then conjugated to Cy5 fluorescent dye to track the enzyme. Conjugation was performed by adding a stock solution of Cy5-NHS ester (Lumiprobe) in dimethylformamide (DMF) to an MPH stock solution to obtain a 2:1 molar ratio with ∼10% DMF. The conjugation process was performed for 4 h at room temperature. Upon completion, the enzyme solution was washed with activity buffer using an Amicon Ultra 15 mL centrifugal filter. The conjugation process was validated by measuring the emission spectrum after excitation at 600 nm using a plate reader (TECAN Infinite M200PRO). The encapsulation process was then performed as outlined above, and images were acquired using a confocal microscope (ZEISS LSM 900, ZEISS Germany) at Ex. 646 nm and Em. 662 nm.

### BocFF-MPH Encapsulation Efficiency Analysis

Initially, BocFF-MPH-Cy5 samples were prepared as outlined above to obtain samples at a concentration of 4 mg/mL peptide and 1 μM of fluorophore-labeled enzyme. The fluorescence of samples was then measured using a plate reader (TECAN Infinite M200PRO) at Ex. 600 nm and Em. 665 nm prior to and after centrifugation at 20,000 rcf for 30 min. The encapsulation efficiency was then calculated as follows
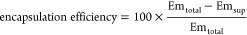
where Em_total_ represents the emission value of the samples before centrifugation while Em_sup_ represents the emission value of the supernatant after centrifugation. Samples were prepared in triplicate and measured twice. Control samples consisted of only BocFF and MPH-Cy5 samples.

### Encapsulated MPH’s Thermal Durability

To test the effect of BocFF encapsulation on the enzyme thermal durability, free MPH and BocFF-MPH were incubated at various temperatures (0–80 °C) for 30 min. Following incubation, the enzymatic activity of 0.025 μM free MPH and 0.025 μM BocFF-MPH toward paraoxon was measured at 25 °C for 10 min, as described above. Spontaneous hydrolysis in samples without the enzyme was used as a control. The activity of MPH at different temperatures is shown as the percentage of the highest activity measured from an average of 3 repeats.

### Encapsulated MPH’s Durability

Free MPH and BocFF-MPH were incubated at 25 °C with shaking (350 rpm) for 132 days using a Thermal block shaker, and the residual activity was tested every several days. The activity of 0.025 μM free MPH and 0.025 μM BocFF-MPH toward paraoxon was analyzed by monitoring the absorbance, as described above. Activity buffer and empty BocFF capsules were used as controls. The presented enzyme activity (mOD/min) is an average of 3 repeats.

### Durability of Free and Encapsulated MPH in Bacterial Culture

Overnight cultures of *Arthrobacter* sp. 4Hβ, kindly provided by Prof. Segula Masaphy, MIGAL—Galilee Research Center, Israel,^51^ were grown at 28 °C. The cultures were suspended and then diluted (1:100) in 2 mL LB medium containing 0.05 μM of either free MPH or BocFF-MPH at different time points after MPH purification and encapsulation (12 and 19 days). The samples were then incubated at 28 °C with 100 rpm shaking for 24 h. Next, 200 μL samples were used to test enzyme activity toward 0.05 mM paraoxon, as described above, at 405 nm. The presented enzyme activity (mOD/min) was calculated from an average of 3 biological repeats, each with 3 technical repeats.

### Hydrolysis Activity of Free or BocFF-Encapsulated MPH with *Arthrobacter* sp. 4Hβ Bacterial Culture

*Arthrobacter* sp. 4Hβ bacteria were grown in 10 mL of LB at 28 °C and 180 rpm overnight, then diluted (1:100) in 200 mL of LB, and incubated under the same conditions. On the next day, cultures were diluted (1:100) in M9 minimal medium.^52^ M9 salts were prepared as described previously,^52^ and then, 200 mL of prepared M9 salts were supplemented with 1 M MgSO_4_ (2 mL), 20% glucose (20 mL), and 1 M CaCl_2_ (100 μL), and the total volume was adjusted to 1L with distilled H_2_O. Next, PNP was supplemented into the solution with a final concentration of 0.115 mM to accustom the bacteria for the consumption of the PNP. The medium was then used to inoculate *Arthrobacter* sp. 4Hβ bacteria in 96-well plates with 0.01 μM of either BocFF-MPH or free purified MPH enzyme (2 and 132 days after purification and encapsulation). The free MPH incubated for 2 days after purification without a bacterial culture was used as a control. Paraoxon at 0.2 mM was added to the enzyme and bacteria mixtures, and absorbance was monitored at 405 nm using a microtiter plate reader (BioTeK, optical length of ∼0.5 cm) at 28 °C for 4 h. Orbital shaking took place before each measurement, and each condition was measured in triplicates.

## Results and Discussion

### Expression, Purification, and Activity Verification of MPH

**Figure 1:**
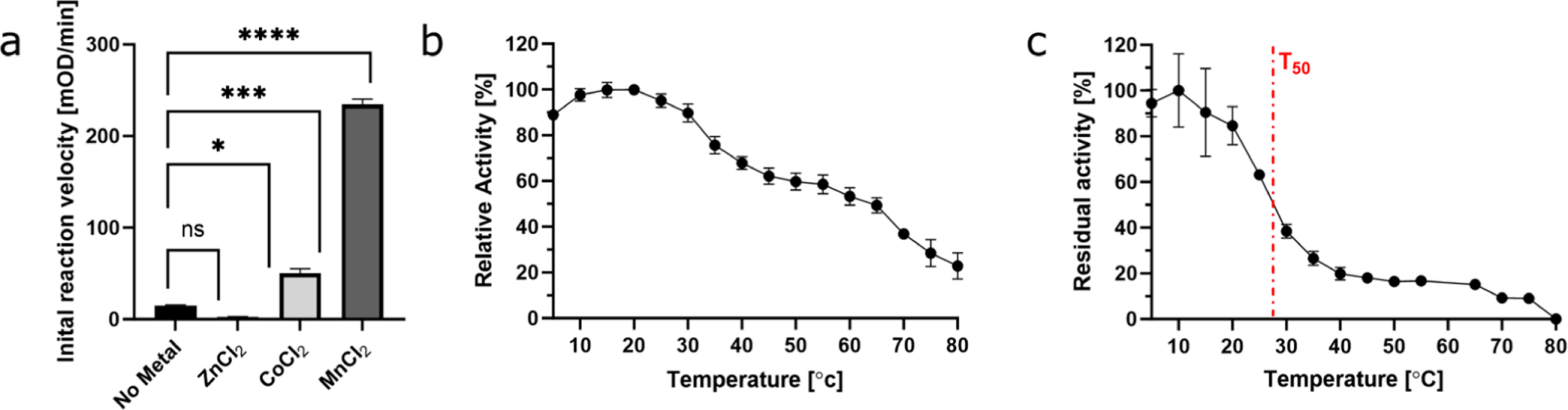
Characterization of MPH activity. (a) MPH activity in bacterial lysates following expression in media supplemented with different metals. The average slope [mOD/min] of the initial reaction velocity was determined using 1 μL lysate with 0.05 mM paraoxon in activity buffer; reactions were performed in triplicates. (b) MPH activity toward paraoxon was tested at different temperatures. (c) MPH residual activity toward paraoxon was measured at the optimal temperature following a 30-min incubation at temperatures ranging between 0 and 80 °C. The red line indicates the T_50_ value at 27.5 °C.

Although MPH is highly efficient toward methyl parathion (1 × 10^6^ M^–1^ sec^–1^), it also shows activity toward other OPs, including paraoxon.^49^ As we aimed to test the ability of conjugating the paraoxon hydrolyzing activity of MPH with bacteria that consume PNP, we explored the best conditions for the enzyme to maintain high activity with paraoxon, in terms of cofactors (metal ions) needed for the activity as well as the temperature range. Supplementing different metals to the growth media during MPH expression can lead to different specificity and an increase in enzymatic activity toward different OPs, as was shown before.^53^ Therefore, in order to identify the preferred catalytic metal for paraoxon degradation, different metal ions were tested. For this purpose, MPH was expressed in*E. coli*-BL21 (DE3) cells in the presence of different divalent metals commonly found as cofactors of the metallo β-lactamase family, namely zinc, cobalt, and manganese.^50,53^ Following bacterial lysis, the highest activity of ∼234 mOD/min was observed in the presence of MnCl_2_ compared to ∼2 and ∼50 mOD/min detected in the presence of ZnCl_2_ and CoCl_2,_ respectively (Figure 1a). These results are in line with a previous study, showing that the phosphotriesterase activity of MPH was higher in the presence of Mn^2+^ compared to Zn^2+^ and Co^2+^.^53^ Subsequently, large-scale expression of MPH with MnCl_2_ supplemented to the culture followed by protein purification was performed, obtaining a highly pure enzyme (Figure S1). Next, the optimum activity temperature and thermostability of the purified Mn-reconstituted MPH were analyzed. The optimal catalytic activity toward paraoxon was obtained at ∼23 °C, and the enzyme maintained >50% of its activity up to 60 °C (Figure 1b). We then tested the residual activity at different temperatures. T_50_ is a stability parameter that expresses the capability of an enzyme to maintain 50% of its activity after incubation at different temperatures. The MPH T_50_ was found to be at ∼27.5 °C as it had lost more than 50% of its activity above this temperature (Figure 1c). In comparison, a recent work showed higher thermal stability of Zn-reconstituted MPH.^54^ This suggests that while the Mn-reconstituted enzyme exhibited increased activity toward paraoxon in comparison to the Zn-reconstituted MPH, particularly in the range of bacterial growth relevant to its conjugation with bacteria, it presents lower stability at higher temperatures. Moreover, after incubation at 28 °C, MPH lost 50% of its activity. These results highlighted the need for enzyme immobilization for increased durability to conjugate it to bacterial culture.

### Characterization of Enzyme Encapsulation

**Figure 2:**
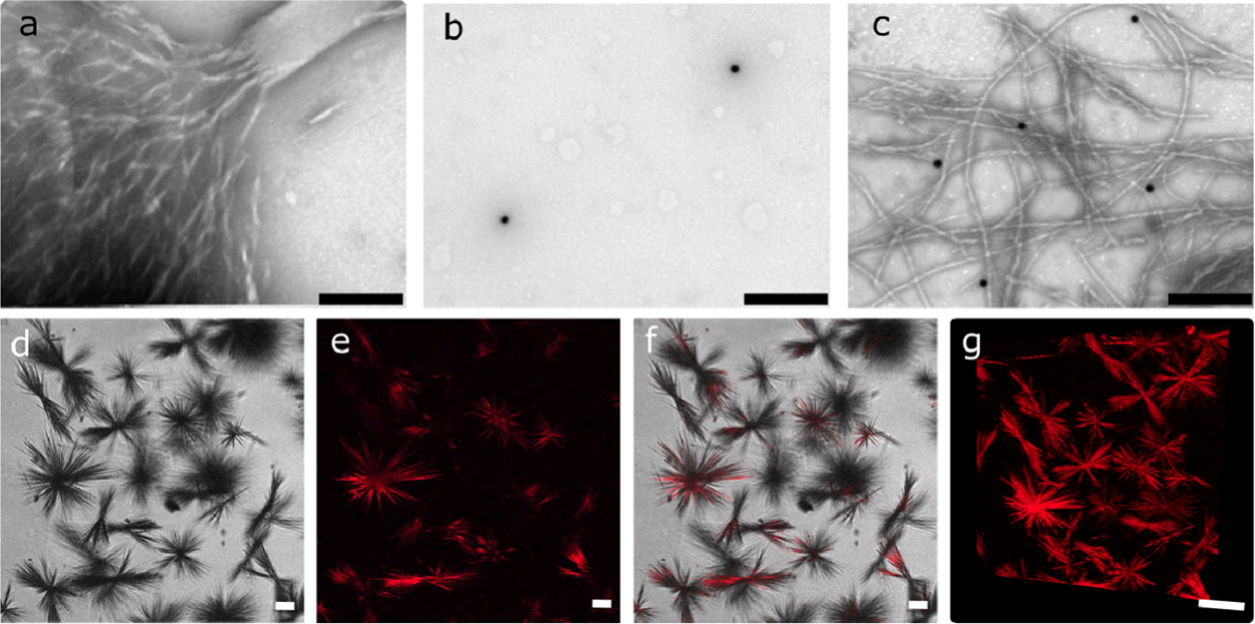
MPH enzyme encapsulation in BocFF fibrils. (a–c) TEM micrograph images: (a) self-assembled BocFF-MPH structures (scale bar 250 nm), (b) MPH-AU_NPs_ particles (scale bar 500 nm), and (c) BocFF-MPH-AU_NPs_ (scale bar 500 nm). (d–f) Confocal microscopy images of Cy5-labeled MPH enzymes encapsulated in BocFF structures: (d) brightfield, (e) fluorescence (Ex. 646 nm and Em. 662 nm), and (f) merged images (scale bar 20um). (g) Z-stacked confocal microscopy image of Cy5-labeled MPH enzymes encapsulated in BocFF fibrils and dispersed in a homogeneous manner (Ex. 646 nm and Em. 662 nm, scale bar 50um).

Enzymes are inherently prone to activity loss due to denaturation caused by extreme conditions, such as high temperatures. We and others have previously shown that enzyme encapsulation could provide stabilization and prolonged shelf life.^31,32,55^ To increase the durability of Mn-reconstituted MPH, purified MPH enzyme was encapsulated within BocFF peptide-based fibrils toward its subsequent addition to bacterial cultures. The BocFF peptide is naturally hydrophobic and, therefore, does not dissolve at room temperature in a buffer solution. However, the high temperature allows for powder dissolution upon heating of the solution. Then, upon cooling the solution, the peptide monomers started to self-assemble. Scheme 1 depicts the preparation process of the self-assembled enzyme encapsulating structures. Initially, the peptide powder is placed into the enzyme activity buffer and then heated to dissolve the peptide into monomers within the solution completely. The peptide monomeric solution is then left to cool in order to allow the assembly of the peptide nanostructures, followed by the addition of the enzyme stock solution while the structures are formed. However, since peptide self-assembly is driven by noncovalent interactions, these might be influenced by the presence of additional components in the solution, such as metal ions, which are present in the enzyme activity buffer. Therefore, we initially performed PXRD in order to assess the effect of the enzyme and buffer on the crystallinity of the peptide. The results revealed minor changes in the crystalline spectra of the peptide, showcasing the effect of the enzyme incorporation into the structure (Figure S2). To note, we assume that the intensity of the peaks in the PXRD spectra of BocFF-MPH is lower than that of neat BocFF due to the crystallization of the salt buffers, which can mask the signal obtained from the peptide. The assembly and validation of the peptide structure formation were then studied using microscopy analysis. TEM images of enzymes encapsulated within BocFF confirmed the formation of nano- and microfibrillar peptide structures, which were formed upon the cooling of the peptide solution, indicating the formation of peptide structures in the buffer (Figure 2a). Analysis of TEM images indicated the formation of peptide nanofibrils ∼23.7 ± 4.5 nm in diameter. To assess the encapsulation of enzymes within the peptide structures, enzymes were tagged using reactive gold nanoparticles (AU_NPs_-NHS ester) and a reactive fluorescent dye (Cy5-NHS ester), allowing their tracking using TEM imaging and confocal microscopy. The successful conjugation reaction of both reactive components was validated by absorbance spectrum measurements (Figure S3). While both MPH and MPH-AU_NPs_ exhibited peaks at ∼280 nm related to the presence of the amino acids, only the MPH-AU_NPs_ exhibited an additional peak at 529 nm, confirming the presence of the AU_NPs_ (Figure S3a–b). Regarding Cy5 conjugation, the pristine Cy5 sample exhibited an emission peak at 663 nm, while the MPH-Cy5 sample exhibited an emission peak at 668 nm, indicating the successful conjugation of the Cy5 dye to the MPH enzyme (Figure S3c). TEM imaging of free AU_NPs_-MPH showcased free-floating particles, which were apart, while the encapsulation process of the AU_NPs_-MPH in BocFF resulted in the presence of the particles on the surface of the peptide fibrils (Figure 2b–c). The results suggest that the particles were present on the surface of the fibrils, possibly due to the attachment of enzymes to the fibrils. Similar to the TEM images, confocal microscopy imaging of empty and enzyme-containing BocFF structures showcased the formation of multiple microscale fibrillar peptide structures in the presence of the buffer solution. No fluorescence was detected in the confocal images of empty BocFF samples (Figure S4). In comparison, the images of the BocFF-MPH-Cy5 samples confirmed the formation of the peptide structures as well as the enzyme encapsulation within the structures, as indicated by the red fluorescence in Figure 2d–g. Although the TEM imaging indicates the presence of the enzyme on the surface of the fibrils, the confocal microscopy imaging suggests the incorporation of the enzymes both on the peptide structure surface and inside the structures in a homogeneous manner (Figure 2g). As mentioned above, the self-assembly of the peptide is known to be driven by the monomers noncovalent interactions. Such interactions are formed not only between the peptide monomers but also between the monomers and the enzymes. Therefore, the enzymes could be absorbed onto the peptide structures surfaces and also incorporated into the structures during their assembly process as a result of interactions between the protein and peptides.

### Durability and Activity of BocFF-Encapsulated MPH in Buffer and Bacterial Cultures

**Figure 3:**
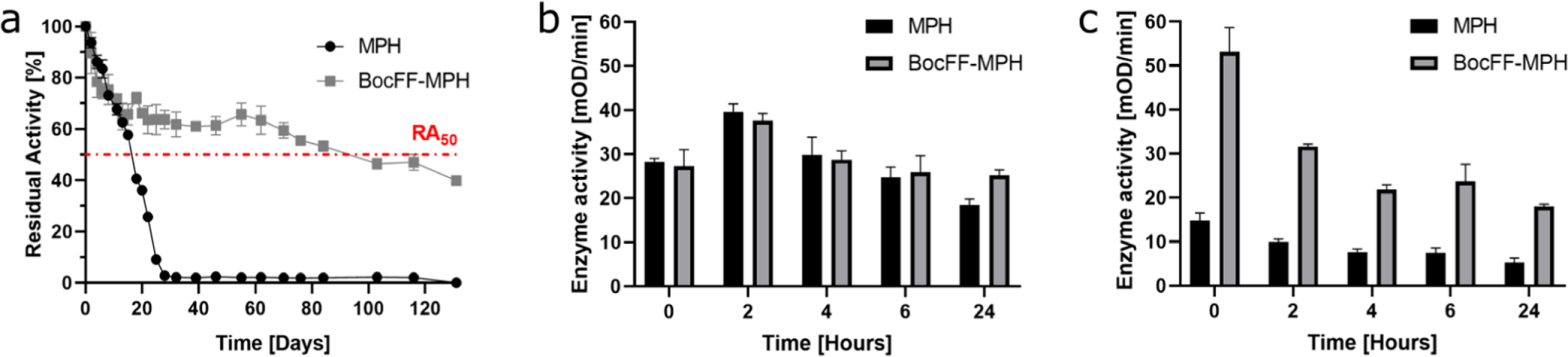
Encapsulated enzyme activity in buffer and bacterial culture. (a) Free MPH and BocFF-MPH activity toward paraoxon over time in activity buffer, at 25 °C. The red line indicates 50% of the activity; for free MPH, the activity reached this value after ∼19 days, for BocFF-MPH, after ∼90 days. (b–c) Enzymatic activity of aged-free and encapsulated MPH after incubation in *Arthrobacter* sp. 4Hβ bacterial culture (b) 12 days and (c) 19 days after enzyme encapsulation.

Following the validation of peptide structure formation and encapsulation of the enzymes, we proceeded to test the enzyme activity. The encapsulated enzyme, BocFF-MPH, maintained ∼70% of activity (151.1 ± 8.7 mOD/min) compared to the free enzyme in buffer (216.4 ± 1.4 mOD/min) (Figure S5). The decrease in activity of the enzyme upon encapsulation at time point zero suggests an effect of substrate diffusion, meaning that the enzyme exhibits lower activity since it is partially immobilized within the structures, requiring the substrate to diffuse inside the nanostructures to be in proximity to the enzyme and reach its active site. Furthermore, no detectable hydrolysis of paraoxon was observed in the control samples, which included neat buffer or empty BocFF fibrils (Figure S5). Encapsulation efficiency was analyzed using the Cy5-conjugated MPH, revealing that ∼28.2 ± 7.0% of the enzyme was encapsulated within the structure (Figure S6). Evaluation of the effect of encapsulation on the MPH’s thermal stability indicated no significant improvement in the stability as a result of the encapsulation (Figure S7). Then, to examine the enzyme’s shelf life, we followed the activity of BocFF-encapsulated MPH compared to the free enzyme. The activity measurements were performed at 25 °C over time. While the free enzyme in the activity buffer lost activity by day 28, the encapsulated enzyme remained active with over 50% residual activity even after 80 days and retained >40% of its activity after 132 days (Figure 3a). Due to the free MPH activity loss by the 28th day, the results might suggest that in the encapsulated system, the free enzyme, which consists of ∼70% of the total enzyme, had lost its activity after 28 days, while the ∼30% encapsulated enzyme maintained function and thus prolonged the enzyme shelf life. Previously, it has been shown that enzyme immobilization leads to the stabilization of enzymes due to their physical confinement within the structures. The physical confinement of proteins within structures and carriers inhibits their local unfolding, which would have ultimately led to the loss of enzymatic activity.^56^ As such, it could be assumed that the encapsulation of the peptides within the BocFF structures prevents them from unfolding, and thus, their durability and activity are enhanced over time. In addition, we examined the activity of free and encapsulated MPH in a PNP-degrading *Arthrobacter* sp. 4Hβ bacterial culture as bacteria secrete extracellular proteases and can change the chemical environment with the potential of inhibiting enzymatic activity. The activity buffer and empty BocFF capsules were used as controls, and their values were subtracted from the enzymatic activity values. At 12 days after enzyme encapsulation, we measured the enzyme activity for 24 h. No significant difference in paraoxon hydrolysis was observed between the encapsulated and free enzymes after incubation in bacterial culture, (Figure 3b). Interestingly, although the activity of the encapsulated enzyme after 19 days was expected to be lower than that of the encapsulated enzyme after 12 days, the results showcased higher activity at the beginning of the measurements after 19 days from encapsulation. This difference in the activity of the encapsulated enzymes could be attributed to the dynamic nature of the supramolecular structures that can lead to changes in the localization of the enzyme molecules in relation to the fibers. Therefore, we hypothesize that the higher enzymatic activity of BocFF-MPH after 19 days of incubation is due to the release of the enzymes from within the structures to the surface or to the medium. Furthermore, after 19 days from enzyme encapsulation, a significant decrease in the activity of the free enzyme was observed, as measured over the time course of 24 h, while the activity of the encapsulated enzyme was higher. Although activity loss occurred for the encapsulated enzyme after 19 days of incubation as well, its activity was 2-fold higher than that of the free enzyme in the bacterial medium even after 24 h (Figure 3c). This suggests that the encapsulation process resulted in increased durability of the enzyme in bacterial culture conditions as well.

### One-Pot Degradation of Paraoxon and PNP by Encapsulated Enzyme and Bacteria

**Figure 4:**
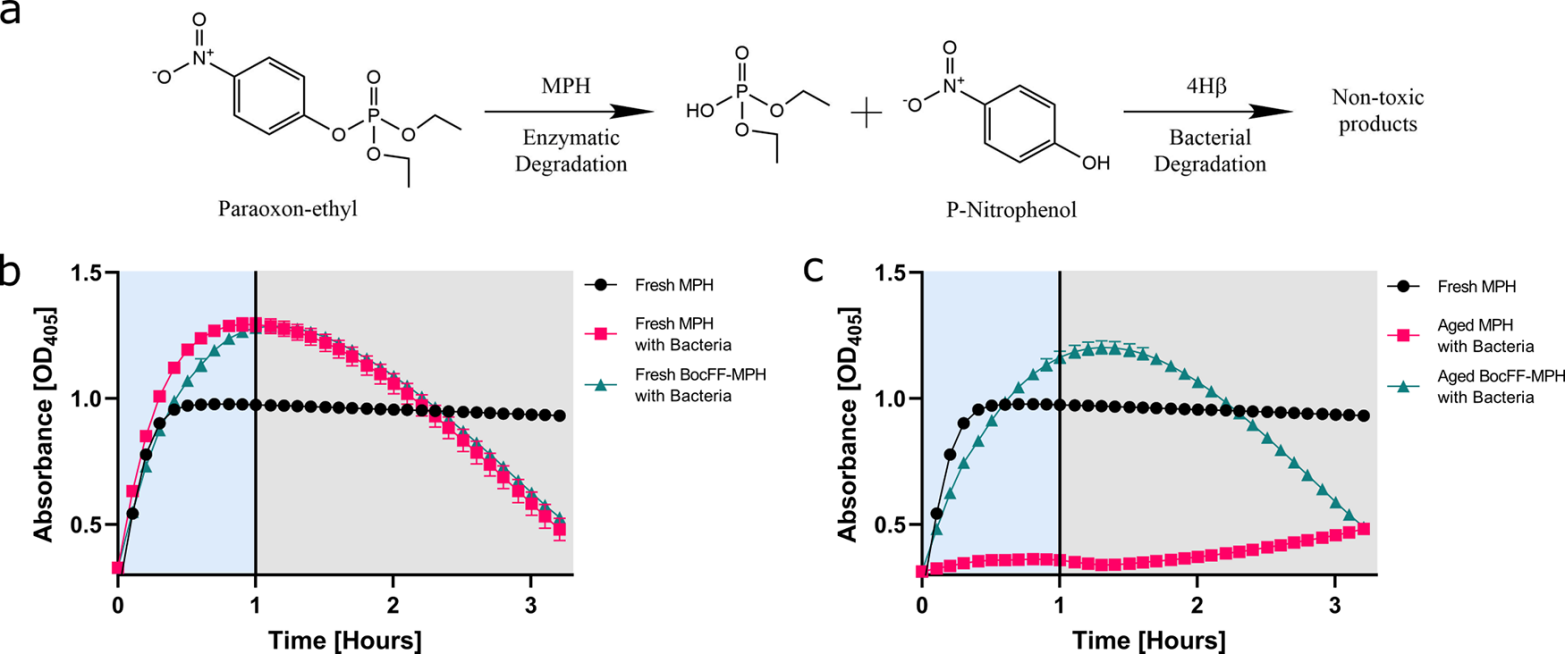
One-pot complete degradation of paraoxon and PNP by enzymatic activity and bacterial consumption. (a) Illustration of the degradation process of paraoxon and enzymatic byproducts. (b–c) One-pot degradation of paraoxon and PNP by free and encapsulated MPH (b) 2 days and (c) 132 days following enzyme purification and encapsulation. The free MPH incubated for 2 days after purification without bacterial culture was used as a control (black). Activity was tested by adding 0.2 mM paraoxon to the enzymes with or without bacteria, and absorbance was measured at 405 nm at 28 °C for 4 h. Measurements were performed in triplicates.

We then continued to assemble a one-pot system for the complete degradation of paraoxon by introducing it to a flask containing encapsulated MPH enzyme with bacteria that can degrade PNP such as *Arthrobacter* species (Scheme 1).^48^ It has been previously reported that *Arthrobacter* sp. 4Hβ is capable of PNP degradation through the production of hydroquinone, which the bacteria then use to obtain carbon, nitrogen, and energy for growth. Tracking of hydroquinone, the intermediate product from PNP degradation by the bacteria, indicated constant and low hydroquinone levels due to its further degradation. Therefore, PNP production and degradation could be robustly monitored using the same wavelength by measuring the increase in absorbance due to the enzymatic degradation of paraoxon by MPH into diethyl phosphate and PNP. Then, the decrease in PNP absorbance correlates with its degradation by native bacteria (Figure 4). Furthermore, we monitored the hydrolysis of paraoxon into PNP by both fresh (2 days after encapsulation) and aged (132 days) encapsulated MPH, and free MPH as a control, in the presence of *Arthrobacter* sp. 4Hβ. In addition, the effect of varying paraoxon concentrations on bacterial growth was evaluated. No significant effect on bacterial growth was detected upon the addition of 0.1 or 0.2 mM paraoxon (Figure S8).

Paraoxon hydrolysis and PNP degradation were analyzed by monitoring the increase or decrease, respectively, in absorbance at 405 nm, as the degradation of paraoxon and of PNP occurs sequentially and not simultaneously in the system, primarily due to the fast kinetics of MPH (Figure 4). This assay was performed for a time course of 4 h. After 2 days from purification and encapsulation, both free and encapsulated MPH presented similar rates of paraoxon hydrolysis over the first hour, 37.7 ± 1.5 and 30.3 ± 0.9 mOD/min, respectively (Figure 4b). Only after 1 h, a reduction in the absorbance was observed due to bacterial consumption and degradation of PNP with a rate of 8.1 ± 0.3 and 7.8 ± 0.1 mOD/min, for the free and encapsulated MPH systems, respectively. Interestingly, after ∼30 min, the samples containing both enzymes and bacteria displayed higher absorbance values than expected in comparison to the samples containing only enzymes. This could be attributed to the formation of intermediate PNP species resulting from a microbial modification of the PNP following the enzymatic step.

Notably, a significant advantage was observed for the aged, encapsulated enzyme (132 days) in comparison to free MPH (Figure 4c). While the free enzyme exhibited a hydrolysis rate of 1.8 ± 0.2 mOD/min in its initial step with a slight increase after 1.5 h, the preserved encapsulated enzyme exhibited a rate of ∼23.9 ± 0.8 mOD/min. The slight increase after 1.5 h in the curve of the aged-free enzyme can be explained by the low promiscuous activity of the enzymes naturally expressed by the bacterial culture. Due to the loss of activity of the free MPH enzyme, only the BocFF-MPH system exhibited the degradation of paraoxon and the formation of PNP followed by PNP degradation. The degradation rate of PNP within the aged BocFF-MPH and bacteria system was ∼8.1 ± 0.1 mOD/min, similar to the rate observed for the fresh enzyme and bacteria system. This suggests that the enzyme aging process and loss of enzymatic activity do not affect the degradation of PNP, only its formation via paraoxon degradation. The results substantiate that the BocFF nanoparticles enhance the durability of the encapsulated enzyme and allow its use even 132 days after encapsulation in a one-put reaction with a bacterial culture that further degrades PNP into nontoxic products at higher efficiency, compared to the free enzyme, in bacterial culture.

## Conclusions

Various organophosphate hydrolases have been characterized for their ability to hydrolyze paraoxon into diethyl phosphate and PNP efficiently.^49,50,57^ However, PNP is considered to be a toxic byproduct, which could, in turn, cause harm to the environment and wildlife.^58^ Several native bacteria, such as *Arthrobacter* sp. 4Hβ, were previously shown to be able to degrade PNP at various efficiency rates.^51,59−63^ Moreover, the engineering of bacteria with the ability of OP degradation and PNP mineralization was shown as well.^64−66^ On the one hand, the application of cell-free, highly active (native or engineered) enzymes is not always sufficient for the complete mineralization of environmental pollutants. Furthermore, the release of engineered bacteria for bioremediation is still not considered a common practice.^67−70^ In this research, a bioinspired one-pot system consisting of an encapsulated enzyme and native bacteria was developed for the bioremediation of xenobiotics such as an OP-based pesticide. To this end, we initially performed large-scale recombinant expression and purification of the highly active MPH with the necessary metal ions as cofactors, followed by biochemical characterization in temperature range, as well as durability analysis in buffer and in the presence of bacterial culture. As with many other enzymes, the purified enzyme was marginally stable and lost more than 50% of its activity after an 18-day incubation at 25 °C. Therefore, we encapsulated the enzyme within BocFF peptide structures, which was confirmed by microscopy analysis. As a result, a significant increase in the ability of the enzyme to maintain activity at room temperature was observed, as the ability to maintain 50% of its activity was increased from ∼15 days to >80 days in the activity buffer. Moreover, higher activity of the encapsulated enzyme compared to the free enzyme was observed in bacterial cultures after 19 days. To achieve full degradation of the pesticides into nontoxic chemicals, a one-pot system was then applied, including the pesticide (paraoxon), encapsulated MPH, and bacteria that consume the toxic byproduct (PNP). In the first hour, paraoxon degradation by the MPH phosphotriesterase activity was dominant, as an increase in the absorbance (corresponding to PNP production) was observed. Next, due to the presence of *Arthrobacter* sp. 4Hβ, a reduction in PNP was detected. We surmise that this one-pot system composed of environmentally friendly components can be applied for the full degradation of the paraoxon pesticide into nontoxic byproducts. We further suggest that such a system, using encapsulated enzymes, can be tailored for different hazardous chemicals, combining different enzymatic activities with native bacteria by first encapsulating the purified rate-limiting key enzymes followed by their mixture with native bacteria, ideally common in the environment of the application, that can use the byproducts for growth. This system can offer another alternative to laborious synthetic engineering of the complete catabolism pathway in bacteria or consortium and relieve the concern of maintaining the stability of these designed functionalities in fluctuating environments.

## Supporting Information Available

Additional experimental results including SDS–PAGE analysis of large-scale MPH purification, PXRD diffraction of BocFF and BocFF-MPH, analysis of Cy5 and AU_NPs_ labeled enzymes, CLSM imaging of BocFF, fresh MPH and BocFF-MPH activity measurements in buffer, encapsulation efficiency analysis, thermal stability analysis of free and encapsulated MPH, and bacterial culture growth curves in the presence of enzymatic solutions (PDF)

## Supplementary Material

am4c06501_si_001.pdf

am4c06501_si_002.mov
