# Supplementary material for: Coupling Peptide-Based Encapsulation of Enzymes with Bacteria for Paraoxon Bioremediation
Source: ACS Appl Mater Interfaces. 2024 Jun 26;16(27):35155–65. doi: 10.1021/acsami.4c06501 (PMC11247427; doi:10.1021/acsami.4c06501)
Supplement: Supplementary file 1 — am4c06501_si_001.pdf [file am4c06501_si_001.pdf]

Supporting Information

Coupling peptide-based encapsulation of enzymes with bacteria for paraoxon bioremediation

Yoav Dan[a, b, c], *David Gurevich*[d], *Ofir Gershony*[d], *Francesca Netti*[a, b, c], *Lihi Adler-Abramovich*[a, b, c*], *Livnat Afriat-Jurnou*[d, e*].

[a] Department of Oral Biology, The Goldschleger School of Dental Medicine, Faculty of Medical and Health Sciences, Tel Aviv University, Tel Aviv, 6997801, Israel. [b] The Center for Nanoscience and Nanotechnology, Tel Aviv University, Tel Aviv, 6997801, Israel. [c] The Center for the Physics and Chemistry of Living Systems, Tel Aviv University, Tel Aviv, 6997801, Israel. [d] Migal-Galilee Research Institute, Kiryat Shmona, 11016, Israel. [e] The Faculty of Sciences and Technology, Tel-Hai College, Upper Galilee, 1220800, Israel.

Emails: lihia@tauex.tau.ac.il, livnatj@migal.org.il

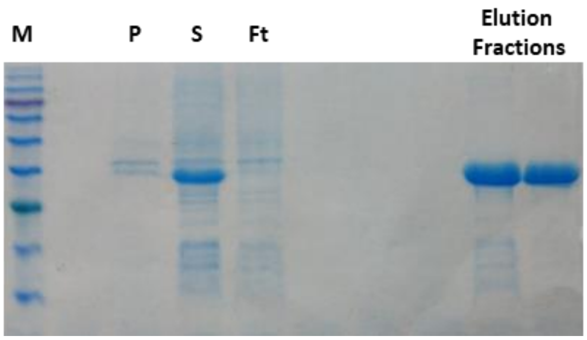

Figure S1. SDS-PAGE analysis following large-scale MPH purification. The lanes are: M-marker, P-pellet, insoluble fraction; S-supernatant, soluble fraction; Ft-flow-through supernatant after passing through a His-trap column.

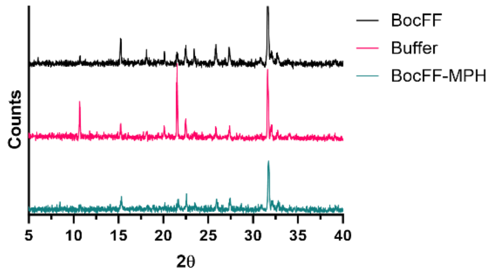

Figure S2. Powder X-ray diffraction of BocFF and BocFF-MPH.

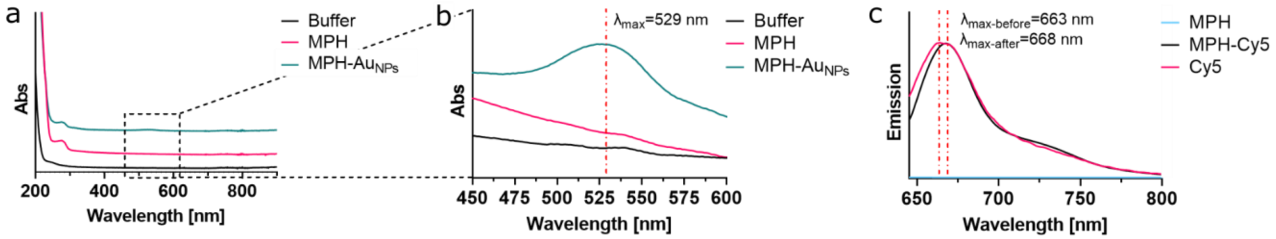

Figure S3. Analysis of Cy5 and AU_{NPs} labeled enzymes. (a-b) Absorbance readings and comparison of free and AU_{NPs} labeled MPH. (c) Emission readings of free and Cy5 labeled MPH.

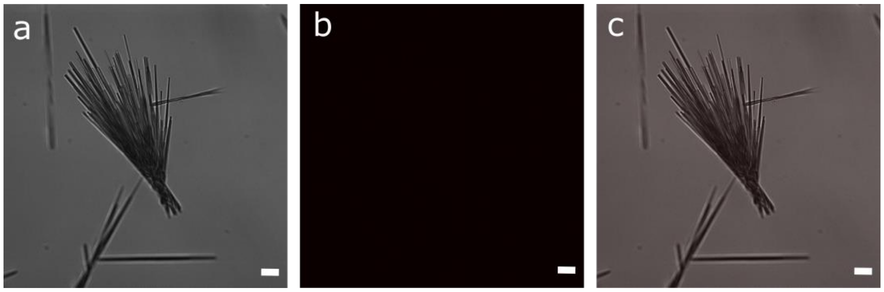

Figure S4. Confocal microscopy images of BocFF fibrils. (a-c) Confocal microscopy images of pristine BocFF structures: (a) brightfield, (b) fluorescence, and (c) merged images (scale bar 20uM).

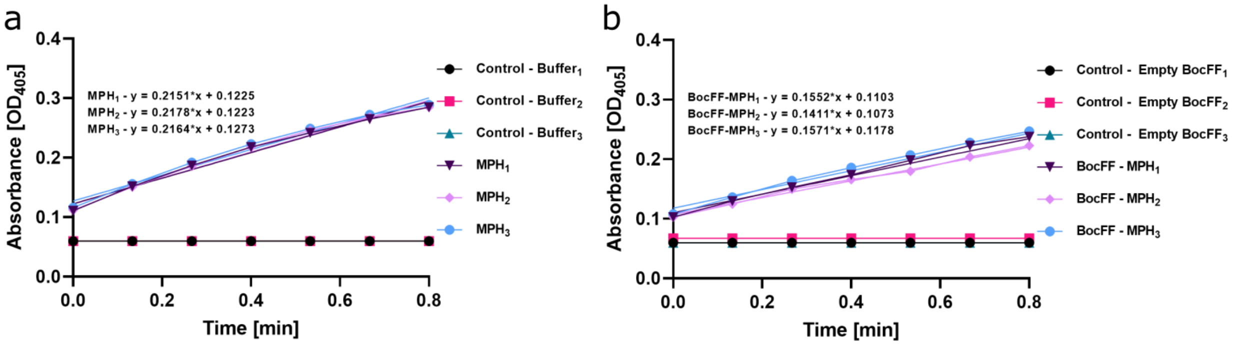

Figure S5. Enzymatic activity of fresh free and encapsulated MPH. The hydrolysis of paraoxon was monitored by following the appearance of PNP at 405 nm. The graphs represent the hydrolysis of paraoxon in (a) buffer solution alone or buffer solution containing the enzyme, (b) buffer solution containing empty BocFF or enzyme encapsulated in BocFF particles. The enzyme activity reaction mixtures contained 0.1 mM paraoxon with 5 nM enzyme in activity buffer (100 mM Tris pH 8, 100 mM NaCl, 0.1 mM $MnCl_2$), the DMSO solvent was less than 2%. Error ranges represent the standard deviation of the data obtained from three independent measurements.

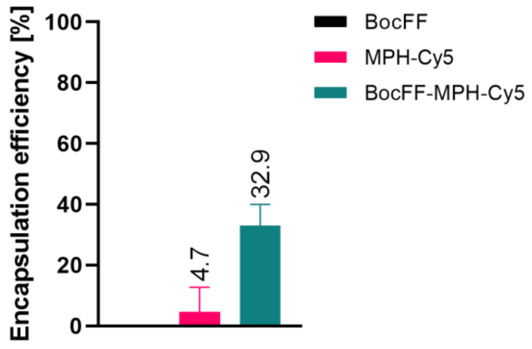

Figure S6. Encapsulation efficiency of MPH-Cy5 within BocFF particles. Encapsulation efficiency values of BocFF particles without the enzyme (black), dispersed MPH-Cy5 (pink), and MPH-Cy5 encapsulated within BocFF particles (green).

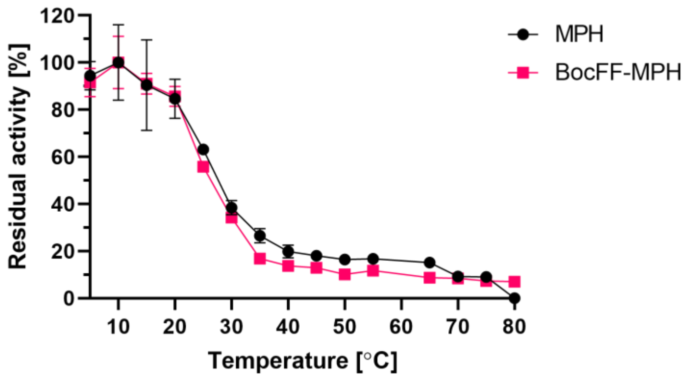

Figure S7. Thermal stability of free and encapsulated MPH. The heat inactivation assay was performed by pre-incubating purified free and encapsulated MPH at temperatures ranging between 0-80° C for 0.5 h. The activity was then measured by monitoring paraoxon hydrolysis at room temperature. The residual activity in percentage was calculated relative to the highest activity.

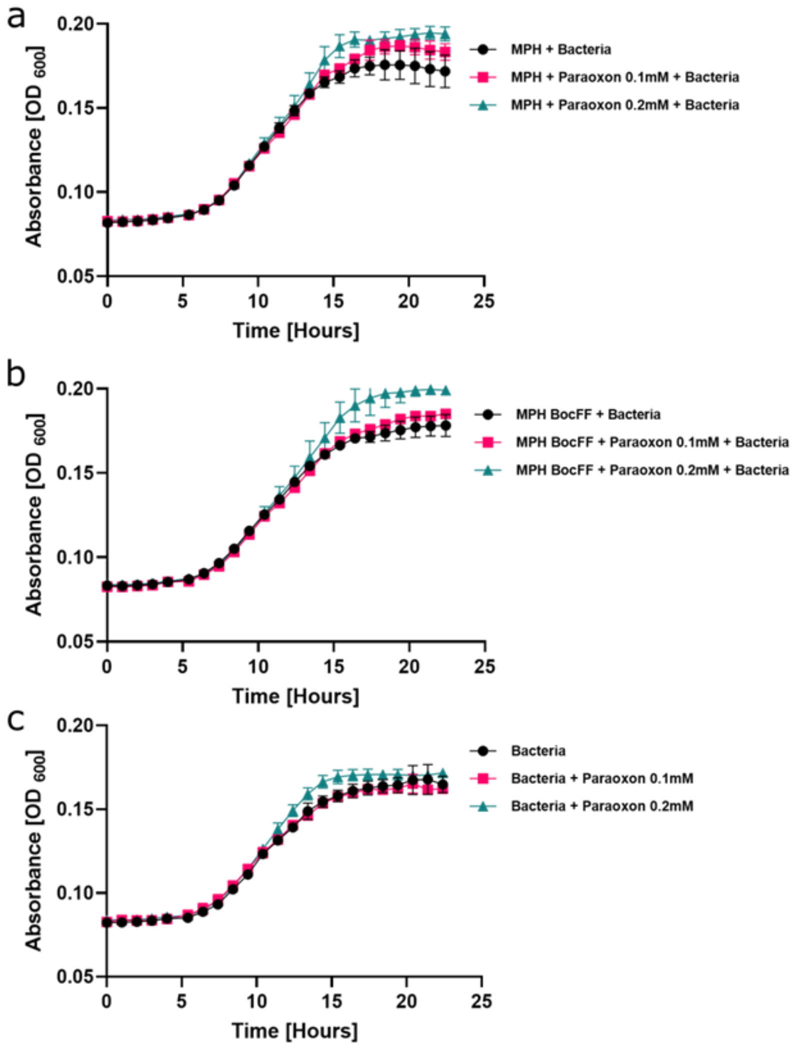

Figure S8. Growth curves of *Arthrobacter* sp. 4Hβ bacterial culture in the presence of enzymes and paraoxon. Growth curves of bacterial cultures at 28°C in the presence of (a) free MPH, (b) BocFF-MPH, and (c) control–only bacteria.
